# How genotoxic is the indoor air environment?

**DOI:** 10.1093/mutage/geag024

**Published:** 2026-06-23

**Authors:** Kirsty Meldrum, Michael J Burgum, Shareen H Doak, Martin J D Clift

**Affiliations:** In Vitro Toxicology Group, Swansea University Medical School, Swansea University, Swansea SA2 8PP, United Kingdom; In Vitro Toxicology Group, Swansea University Medical School, Swansea University, Swansea SA2 8PP, United Kingdom; In Vitro Toxicology Group, Swansea University Medical School, Swansea University, Swansea SA2 8PP, United Kingdom; In Vitro Toxicology Group, Swansea University Medical School, Swansea University, Swansea SA2 8PP, United Kingdom

**Keywords:** genotoxicity, indoor air pollution, particulate matter, volatile organic compounds, metals, gases

## Abstract

The indoor environment changes based on a variety of different factors, therefore changing the genotoxic potential of that environment. Despite the associations of exposure to outdoor air pollution and links to health impacts, enough is not currently known about the impacts to human health indoor air may have. Due to the variable nature of the indoor environment (multiple things can change this environment, including but not limited to, the use, occupants, materials within the environment, animals, the outside environment and ventilation) this can prove difficult. Indoor particulate matter (PM) of various sizes (PM_2.5_–PM_10_ for example) has been shown to induce reactive oxygen species, increase markers in oxidative stress, double strand DNA breaks, and cause 8-oxoguanine lesions in both *in vitro* and *in vivo* models (including humans). The particle type and source influence these responses, highlighting heating and cooking fuel sources that may be more genotoxic than others. A similar pattern has also been observed with volatile organic compounds (VOCs) with various mixtures of VOCs and different VOC compositions inducing DNA damage differently, with diet also being a contributing factor to genotoxic potential. A significant knowledge gap in the potential genotoxicity of indoor air pollution is the exposure of various biologicals found indoors. Moulds, other fungi, and allergens are an emerging health risk and are of significant public interest. Little is known about the inhalation toxicology and mechanisms of action of these components, and this is becoming increasingly important to understand due to public deaths linked to their exposure. There is overlap with the health effects of outdoor air pollution, where there has been much more research focus. Therefore, the aim of this review is to establish what is already known, the potential limitations of the current work and where we should focus research in the future.

## Air pollution

Air pollution is a global concern, with the World Health Organization attributing it to 7 million deaths annually [1], it is also not a new problem with the health effects still requiring research and understanding. Air pollution is composed of various components which are dependent on the location and use of the area (e.g. agriculture and the industry present). Air pollution can be comprised of particulate matter (PM), gaseous compounds, volatile organic compounds (VOCs), polycyclic aromatic hydrocarbons, and biologicals (pollens for example), but in isolation it is yet to be distinguished as to which component is driving the differential toxicology of the measured adverse health effect [2–4]. As well as these primary sources, there is also the impact from aged aerosols and reactions with atmospheric oxidants causing the transformation of primary aerosols into secondary organic aerosols (SOAs), which have been identified as more toxic [5]. When combined with PM it has also been identified that these exposures had more effects when compared to the particles alone [6]. The geographical region where the pollution is produced also influences the characteristics of the pollutant mixture, such as heavy industrial areas, ports and transport hubs, and volcanic activity. Therefore, multiple samples are required to be taken globally to determine which characteristics relate to potential adverse health outcomes and effects [7–11]. The WHO Global Air Quality Guidelines (2021) has highlighted the need to understand the complex mixture of pollution components and the influences that other components (such as chemicals, metals, various gases, and biological elements) have on human health. Under regulation, sulphur dioxide, nitrogen dioxide, and oxides of nitrogen, PM_10_ and PM_2.5_, lead, benzene, and carbon monoxide are required to be monitored (The Air Quality Standards Regulations 2010). With the smallest of the particles, the ultrafine particle fraction (particles <0.1 μm), unable to be monitored without using a surrogate measurement system such as a particle number counter [12]. It is already known that outdoor air pollution and traffic-related air pollution can lead to DNA methylation [13, 14], with location influencing this adverse health impact, even from before birth [15].

As the outdoor environment can also impact the indoor, this gives insight into the potential mechanisms of DNA damage induce by air pollution. It is unclear whether the concentrations or the origin of the components are driving the effect of these mechanisms and therefore if indoor and outdoor can be linked, as there is a distinct lack of the genotoxic information linked to the components of indoor air.

## Indoor air environment

Indoor air pollution (IAP) encompasses any indoor environment with potentially vast differences in the compositions of the IAP matrix. With the average person spending *ca.* 87% of the time within such environments [16] the potential for this exposure to cause health effects should be considered. This includes public areas of education (schools, nurseries, and universities), work and social places (offices, hospitals, shops and supermarkets, and gyms) private housing stock [17], as well as transportation (buses, trains, and cars). IAP is made up of a range of different PM sizes (PM_10_—particles smaller than 10 μm and PM_2.5_—particles smaller than 2.5 μm), VOCs, NO_2_, O_3_, SO_2_, CO*_x_*, various heavy metals, and allergens and microorganisms [18]. The IAP cocktail can be affected by several different factors, with three main contributors being: the circumstance (i.e. cooking [19, 20], cleaning, personal hygiene [21]) within the indoor space (homes will have a different composition to other indoor spaces [22]); the outdoor air [23] (and the boundary effect); and time of year [24]; how the buildings are constructed (as well as the materials used), their ventilation (ventilation systems and opening of windows), the room layouts, and finally what is present within the indoor space [25, 26]. Various components of IAP have already been linked to a range of different health effects and morbidities, including reproductive, cognitive, and pulmonary health [27–29], with links between respiratory disorders and inflammatory pathways documented [30] and with indications that IAP can induce genotoxicity and trigger initiating events such as oxidative stress (OS) [31].

The aerosol transformation process that occurs in the outdoor setting is also a factor in the indoor environment, with reactions with surfaces of materials and the occupants of a space playing a larger part in the changes of chemistry indoors compared to the atmospheric oxidants outdoors. The indoor chemistry of an environment also changes depending on season, time of day, the building type and materials, as well as its geographical location, highlighting again the variability that can be found within indoor environments [32]. Human behaviour further influences the generation and composition of SOAs [33]. These factors are an important consideration with investigating the toxicology of the indoor environment.

There are currently no studies that identify, measure, or examine the effects that various biological components of the indoor environment may have on the genotoxic potential of IAP. This includes various allergens, fungi, and mould which have become apparent as significant health risks within our environment highlighted by the 2024 Ministry of Housing, Communities and Local Government guidance report ‘Understanding and addressing the health risks of damp and mould in the home’ [34]. These gaps are however beginning to be investigated by the IDEAL Consortium [35].

### Particulate matter (PM)

The measurement of PM is relatively low cost and accessible [36], with public mitigation techniques focused towards reducing the PM concentration indoors due to the accessibility of air purifiers and filters [37]. However, the majority of indoor PM is linked to outdoor PM levels [38], and there have been multiple studies linking outdoor PM and genotoxic potential [39–41], it is well established that the indoor environment is significantly different and indoor PM in isolation has also been found to induce genotoxicity (Table 1).

**Table 1:** Table summarising the genotoxicity of PM and metals found within indoor air from various indoor sources.

Model type		Component	Assay utilised	Outcome	Ref
*In Vitro*	*A549*	Wood smoke particles	ROS formation, HO-1 expression	Significant dose dependant increases in ROS after 3 h. No change in the protein concentration of HO-1, but a decrease in expression after 24 h	[43]
*In Vitro*	*HpAEpiC*	solid fuel combustion particles	ROS formation	Significant increase in ROS production as well as mitochondrial damage	[44]
*In Vitro*	*A549*	Ship refit operations particles	ROS formation	There were significant increases in ROS at different stages of the refit processes as well as after certain lengths of exposures	[45]
			MN assay	There were significant increases in micronuclei frequency at different stages of the refit processes as well as after certain lengths of exposures, these samples also increased ROS formation	
*In Vitro*	A549	Candle combustion particles	ROS formation	There was an increase (non-significant) in ROS formation after exposure to the candle particles	[46]
*In Vivo*	Mice—C57BL-6n		FPG comet assay	There was no change in the DNA strand breaks within the lungs after candle particulate exposure	
			Comet assay		
*In Vitro*	Endothelial progenitor cells	Fine and ultrafine particulate	ROS production	There was a significant increase in ROS production in monocytes and granulocytes when compared to lymphocytes	[48]
*In Vitro*	*BEAS-2B*	polystyrene microplastics	ROS production	There was an increase in ROS generation after NH2-PS-MPs exposures compared to PS-MPs-or COOH-PS-MP. Charge influenced the response	[49]
*In Vitro*	*A549 and THP-1*	Cow stable dust	Oxidative Stress	Significant increase in oxidative stress (even between different farms assessed), as well as a significant decrease in the metabolic activity	[47]
*In Vitro*	*A549*	Urban and rural indoor dust	NO production	All the microbes identified within the PM induced NO production	[66]
*In Vivo*	Rat—Wistar Kyoto	Fine PM	HO-1 expression	There was an increase in the expression of HO-1 in a concentration dependant manner in the heart tissue but not the lungs	[55]
*In Vivo*	Rat—Sprague Dawley	Traffic particles	MDA and GSH	There were significant reductions of plasma GSH and significant increases of MDA in maternal and offspring after exposure to the PM	[59]
*In Vitro*	*Salmonella typhimurium* strain TA98 and TA100	PM_2.5_, CO, NOx	Ames test	Stove type and fuel pellet type influences the mutagenic potencies. In order of mutagenic potency: Xuanda stove; peanut husk > Philips stove; hardwood pellets > Xunda stove; hardwood pellets > Mini Moto; peanut hull = Mini Moto; hardwood pellets	[132]
*In Vitro*	*A549* and *THP-1*	Wood combustion smoke and ambient PM_2.5_	Comet assay	Significant increases in SB in the wood smoke PM compared to the ambient background collection. The high oxygen sample significantly increased SB in the THP-1 cells	[69]
			OG	All PM increased the levels of OG; there were no changed in etheno-adducts or bulky DNA adducts	
*In Vitro*	*A549*	Subway and urban street PM_10_	Comet assay	Subway PM were approximately eight times more genotoxic (10, 20, and 40 μg/cm^2^) than the urban street PM	[70]
*In Vitro*	Biomass and LPG fuel use at home (Lucknow City, India)	PM	MN Assay	Elevated MN frequencies in cow dung, then cow dung/wood and then wood alone. Kerosene increased the frequency but not significantly	[79]
*In Vitro*	Home/kitchen—two different fuel sources (Hooghly, Howrah, Burdwan and Birbhum, West Bengal, India)	PM_10_ and PM_2.5_	ROS in leukocytes and SOD in erythrocytes	There was a significant decrease in levels of SOD in the LPG group compared to the biomass group. There is a significant increase in the ROS in the biomass group compared to the LPG group	[50]
*In Vitro*	Asthmatics	PM, CO_2_, and NO_2_	Genes related to OS (HMOX1)	There were no changes in the genes related to DNA repair or oxidative stress after exposure to either candles or cooking	[74]
			Comet assay	Neither cooking nor candle exposure had significant effects on the levels of DNA SB	
			FPG comet assay		
*In Vitro*	Home/Kitchen—two different fuel sources (West Bengal, India)	PM_10_ and PM_2.5_	ROS and SOD in sputum cells	Increase in ROS and a decrease in SOD in biomass users compared to the LPG users	[51]
			Comet assay	Increased DNA damage was recorded in both inflammatory cells (leukocytes and alveolar macrophages) and airway epithelial cells in sputum of biomass users compared to LPG	
*In Vitro*	Home/Kitchen—two different fuel sources (Biomass and LPG) (West Bengal, India)	PM_10_ and PM_2.5_	ROS and SOD in sputum cells	OGG1 expression was localised to the airway epithelial cells, but not the immune cells. There was a positive correlation of ROS generation and PM_10_ and PM_2.5_	[52]
			Comet assay	Biomass use significantly increased the DNA strand breaks when compared to the LPG groups	
			8-oxoG protein analysis	8-oxoG was significantly higher in Biomass fuel users when compared with that of LPG users	
*In Vitro*	Chemical store keepers	PM (various sizes) SO_2_, NO_2_, NH_3_, CO, and O_3_	H_2_O_2_, GSH	The levels of GSH varied between the different stores but were significantly less than the control groups. H_2_O_2_ levels were significantly higher in the shopkeeper group compared to the control	[133]
*In Vitro*	Taxi drivers	Not specified	MDA	MDA concentrations are significantly higher in the taxi group compared to the office workers	[60]
*In Vitro*	Human exposures	PM_2.5_ and ozone	Comet assay	Exposure to house dust or ozone did not change the genotoxicity in PBMCs. Dust+Ozone reduced the total genotoxicity measured	[73]
			FPG comet assay	Exposure to dust+ozone decreased the levels of oxidized purines in PBMCs	
*In Vitro*	Home/kitchen—two different fuel sources (Lucknow, India)	PM	Comet assay	Significantly increased DNA strand breaks in the biomass group when compared to the LPG group (regardless of age)	[72]
*In Vitro*	Biomass and LPG fuel use at home (West Bengal, India)	PM_2.5_ and PM_10_	y-H2AX	Significantly higher expressions of DSB marker y-H2AX in biomass compared to LPG	[78]
			MN assay and comet assay	Micronucleate cell frequencies were significantly higher in the biomass group compared to the LPG group. 59% increase in DNA strand breaks in the biomass group compared to the LPG group	
*In Vitro*	Aluminium factory workers	Aluminium in dust	SOD activity	Different regions within the factory led to different aluminium concentrations, with casting smelters having the highest SOD activity	[122]

PM, particulate matter; ROS, reactive oxygen species; DDIT4, DNA-damage-inducible transcript 4; HO-1, heme oxygenase 1; iNOS, inducible nitric oxide synthase; NO, nitrogen oxide; PS-MPs, polystyrene microplastics; PBDEs, polybrominated diphenyl ethers; VOCs, volatile organic compounds; TRAPA1, transient receptor potential ankyrin 1; ELC PCB, environmental lower-chlorinated PCBs; A549s, human epithelial cell lines; THP-1, macrophage like cell lines; BEAS-2B, bronchial epithelial cell line; Calu-3, epithelial lung cells; LPG, liquid petroleum gas; MDA, malondialdehyde; GSH, glutathione; SOD1, superoxide dismutase 1; SOD2, superoxide dismutase 2; COX-2, cyclooxygenase-2; BAL, bronchial lavage fluid; CO, carbon monoxide; T-AOC, total antioxidant capacity; Ref, reference.

It has previously been established that the majority of endogenous DNA damage occurs from DNA reacting with various water and reactive oxygen species (ROS) within cells [42]. Therefore, it is an important marker that could indicate the potential for IAP to induce DNA damage. Using *in vitro* models, it has been established that exposure to indoor particles or dusts found within indoor environments induces a significant increase in ROS production. Exposure to wood smoke [43], solid fuel combustion [44], ship refitting processes [45], and candle combustion particles [46] in various different lung cells all induced an increase in ROS measured. This includes *in vitro* exposure to cow stable dust [47], fine and ultrafine particulate [48], and polystyrene microplastics [49]. This was also identified in sputum cells from humans after exposure to biomass fuel burning [50].

Particles from different fuel sources (such as biomass burning and liquid petroleum gas (LPG) use) induce different concentrations of ROS. Women that used biomass sources for cooking had a significantly higher ROS concentration when compared to a group using LPG [50–52]. Indicating that regardless of particulate size, there (PM_2.5_ and PM_10_) is the potential for indoor PM from a variety of different sources to stimulate the production of ROS.

Heme oxygenase-1 (HO-1) is an enzyme that catalyses cellular heme to free iron, carbon monoxide, and biliverdin. It is linked to normal physiological processes as well as induction after exposure to various cellular insults [53]. Within normal cells, increase in HO-1 expression has been identified as preventing DNA damage [54]. After exposure to wood smoke particles (indoors), not only was there a dose dependant increase in ROS, but there was a significant decrease in the expression of HO-1 in A549 cells [43], further suggesting that exposure to these specific particulates have the potential to induce DNA damage. Within a rodent *in vivo* model (rats), it was identified that it was an organ specific increase in HO-1, cardiovascular system had increased HO-1 expression in a dose dependant manner; however, the airways had no change in the expression of HO-1 after collected fine PM exposure [55].

Malondialdehyde (MDA) is a biological marker for OS and is most commonly used for a variety of different health conditions [56]. It is an exogenous product of lipid peroxidation [57] and is known to form stable adducts to deoxyguanosine and deoxyadenosine and induce DNA damage [58], this adduct stability has the potential to influence the toxicity [57]. After indoor exposure to outside traffic particles (mimicking being next to an open window for example) in a Sprague Dawley model, significant increases in MDA were found in the plasma of maternal rats, as well as the pups the dams were pregnant at the time of exposure [59]. This can also be identified within human studies with the concentration of MDA in the blood of taxi drivers significantly higher than office workers and those exposed to less unspecified PM [60]. Taken together, this further supports the potential for MDA to act as a biomarker for oxidative damage induced by IAP exposure.

Glutathione (GSH) is a key molecule for cellular homeostasis and is a main defence against oxidative damage that can occur within a cell as it is found in the cytoplasm of all cells [61]. It is predominantly found in a thiol-reduced form within the cells and transport between the cellular compartments is vital to reduce the effects of ROS, and enable redox signalling [61]. It is also involved in DNA repair, cell proliferation and development, and cell death processes. It provides and contributes to the correct redox environment for DNA replication [62], and therefore any dysregulation of this molecule has the potential to lead to DNA damage and mutagenesis. In pregnant rats exposed to indoor exposure to outside traffic particles, as well as having an increased plasma concentration of MDA, this was coupled with a significant decrease in the plasma concentrations of GSH in both the pups and the dams [59]. This is a pattern that is replicated with all the rodent studies that identify an increase in MDA, also observe a decrease in the GSH concentration [63].

There is evidence that the production of nitrogen oxide has the potential to inhibit multiple DNA repair enzymes [64]. Nitric oxide has the capability to interact and damage DNA through a variety of different mechanisms [65]. Using A549 cells exposed to PM, there was an indication that it may not be the particles themselves that stimulate NO production within the model, but the various microbes that are associated with them [66].

The comet assay is used to detect DNA strand breaks (SBs) in the nuclei of cells which have been exposed to genotoxic compounds [67]. It has also been identified as a tool for the biomonitoring of humans exposed to various chemicals (environmentally and occupationally) [68]. After *in vitro* exposures to various PM (from wood smoke and subways—both as indoor components), the comet assay identified the genotoxic potential of wood smoke [69] and subway PM [70] compared to ambient air. As well as being utilised as an *in vitro* assay, it can also be used as a biomarker with whole blood [71]. It has been successfully used in determining the difference in mutagenicity potential between different fuel types, showing that users of biomass fuels have more SBs that those using LPG fuels [51, 52, 72]. These differences can be associated with the fuel types and not age of the user and have been identified in different cell types as well as blood [51]. However, not all exposures within human *in vivo* studies can identify differences in the genotoxic potential of different exposures [73, 74].

The micronucleus assay (Mn Assay) has been utilised as the gold standard for identifying the potential for a substance to cause chromosomal damage, which can be either aneugenic (loss of an entire chromosome) or clastogenic (chromosomal breaks, deletions, or rearrangements) in nature [75]. It can be used for establishing other DNA events by counting the nucleoplasmic bridges (DNA mismatch repair and potentially telomere end-fusions) or nuclear buds (removal of DNA repair complexes) for example as well as scoring the number of micronuclei in the cytoplasm of interphase cells, and cell death (necrosis and apoptosis) [76, 77]. For the exposure of IAP PM, this assay has been utilised for both *in vitro* assessment and human *in vivo* assessment of the potential DNA damage induced via exposure. The frequency of micronuclei in ship construction personnel was found to be a DNA damage indicator which could differentiate between the different ship refit stages (including the PM composition) and which refitting stage has the potential to induce the most damage *in vitro* [45]. This could therefore lead to regulation and additional personal protection equipment required for these refitting processes. It can also indicate difference genotoxic potentials of different fuel types, with those cooking with biomass fuels having a higher micronucleus frequency than those using LPG fuels [78], as well as being able to rank fuel sources [79].

Typically, the verification of double SBs is to use the micronucleus assay, however, follow-up analysis with γH2AX staining and fluorescence imaging can be used as an additional screening tool [80]. Phosphorylation of H2AX produces γ-H2AX, and this process can be exploited as a sensitive marker for DNA double SBs [81]. These events are a representation of the most severe types of DNA damage and can impact genomic integrity [82]. After exposure to biomass PM compared to LPG PM, women using this fuel to cook with had a significantly increased expression of γ-H2AX [78]. This suggests that these women facing an increase in potential DNA damage when inhaling these specific PM. In a 2022 study, it was demonstrated that PM_2.5_ under specific *in vivo* administration (intratracheal instillation) at high-level, short-term can cause DNA damage. The authors did report in addition that the DNA repair pathways were sufficient to alleviate the effect [83].

In several recent studies, PM including PM_10_ and PM_2.5_ have both been implicated in causing 8-oxoguanine lesions in A549 and HaCaT keratinocytes [84–86]. The PM_2.5_ has been shown to cause the 8-oxoguanine lesion in two other key cell types; BEAS-2B and 16HBE14o^−^ [87, 88]. All studies completed with the formamidopyrimidine-DNA glycosylase (FPG)-modified comet assay established that the exposure to PM [46, 73, 74] did not cause an increase in 8-oxoG, they also did not cause an increase in SBs. The 8-oxoG protein levels can also be assessed after exposure to PM. This identified an increase in 8-oxoG protein in biomass fuel users compared to LPG fuel users, agreeing with the comet assay and the potential for the SBs seen in these women to be associated with ROS [52].

Taken together, there are various markers that can be utilised to determine the potential genotoxic potential inhalation of PM may have. This includes different sources as well as different concentrations potentially helping to influence removal of specific fuel sources, changing to alternatives or education on how to reduce the potential exposure to PM capable of inducing genotoxic effects. From the studies outlined above, it is evident that any PM inhaled has the potential to (or begin to) induce genotoxic effects, the level of effect is source and concentration dependant.

### Chemicals and VOCs

As there are no official indoor regulations within the UK for individual VOCs, the Department for Education Guidance BB101: Ventilation, thermal comfort and indoor air quality (DfE, 2018) recommends the limits applied by WHO guidelines for indoor air quality: selected pollutants [89]. There are more studies completed on the various VOCs found within the indoor environment in *in vitro* and *in vivo* rodent models compared to the PM components and their genotoxic potential in the last 20 years. The only human studies completed, where only a VOC is measure, are currently occupational studies (Table 2).

**Table 2:** Table summarising the genotoxicity of VOCs from various indoor sources.

Model type		Component	Endpoint analysed	Outcome	Ref
*In vitro*	Occupational—pathology ward	Formaldehyde	CA	There was a significant (about threefold) increase in the frequency of CA/cell and the percentage of cells with aberrations	[117]
*In vitro*	Occupational—pathology ward	Formaldehyde	M1-dG	Significant increase in adducts in those working in the reduction room compared to the others	[103]
*In vitro*	Occupational—plastic laminate plant	Formaldehyde	M1-dG	Significant increase in the M1dG adducts in the workers compared to control, significantly higher in groups exposed to more than 66 μg/m^3^	[104]
*In vitro*	*Calu-3 and 16HBE14o-*	Formaldehyde	ROS production	Highest concentrations (1400–7000 μM) induced significant ROS production. Cell types influenced the response (Calu-3 significantly higher compared to 16HBE14o-)	[90]
*In vitro*	*THP-1*	Formaldehyde	NO production	Increased NO intracellular levels and secretions after 50 and 100 μM exposures. iNOS was also stimulated by formaldehyde exposures	[106]
*In vitro*	*A549 and NCI-H357*	PBDEs	ROS production	Significant increases in ROS production at the top concentration (45 μg/cm^2^), after exposure to certain PBDEs	[91]
*In vitro*	*Flp-In 293 cells (expressing mutant TRAPA1)*	VOCs—2-ethyl-1-hexanol (2-EH), Texanol, and TXIB	TRAPA1 activation	2-EH and Texanol activated TRAPA1 in a concentration dependant manor	[134]
*In vitro*	*HepaRG, MCF-7, and A549*	ELC PCB congeners	Oxidative stress	Only 1 PCB was found to significantly increase ROS production	[92]
*In vitro*	*A549*	VOCs	Comet assay	No changes in genotoxicity after exposure to a-pinene; Δ3-carene; or hexanal. There were significant increases in genotoxicity after exposure to 100 and 250 and 40 and 250 mg/m^3^ 2-heptenal and 2-octenal respectively with no changes in cytotoxicity	[115]
*In vitro*	Human peripheral blood mononuclear cells	2,4,5-TCP, PCP, 4,6-DCG, TeCG, 4,5-DCC, and TeCC	Comet assay	PCP, TeCG, and TeCC in a dose-dependent manner induced purines oxidation in DNA. The strongest oxidative purines damage was observed for TeCC (1 and 5 μg/ml) provoked significant bases lesions. The 2,4,5-TCP at 1 and 5 μg/ml caused stronger oxidative damage to pyrimidines. Oxidative DNA damage provoked by 4,5-DCC was similar to 4,6-DCG	[135]
*In vitro*	*A549*	Saturated aliphatic aldehydes	DNA methylation	54 genes were commonly methylated compared to in control cells	[136]
*In vitro*	*HaCat*	Airborne PCBs	Telomerase activity and telomere length	All PCBs significantly reduced telomerase activity compared to control (from day 18). Different PCBs induced differences in telomere length (day dependant)	[99]
*In vitro*	*Salmonella typhimurium* strain TA98 and TA100	Α-pinene	Ames test	No mutagenicity was observed in *S. typhimurium* strains TA100 and TA98 after α-pinene exposures. α-Pinene oxide was mutagenic in these same bacterial strains, when tested over a range of 25–500 μg/plate in the absence of induced rat liver S9. With S9 α-pinene oxide was 4-10× more mutagenic than without S9	[137]
*In vitro*	*S. typhimurium strain YG1041*	Aromatic amines, nitroarenes, and PAHs	Ames test—urine	Laibin smoky coal uses had higher urinary mutagenicity compared with smokeless coal users. Diesel exhaust engine-exposed factory workers had significantly higher urinary mutagenicity levels compared to unexposed controls	[138]
*In vivo*	Rat—F344	Hexanal	microarray	Genes associated with apoptosis due to DNA damage (DDIT4) were downregulated in response to exposure	[97]
*In vivo*	Rat—Wistar	Cooking oils	ROS, MDA, and GSH	ROS and MDA were significantly increased and GSH was decreased after the exposures (2 and 20 mg/kg)	[63]
*In vivo*	Mice—Kunming	Formaldehyde, benzene, toluene, xylene, and CO	T-AOC, GSH-Px, and SOD activity	There were no significant differences in MDA and SOD levels. T-AOC and GSH-Px showed that there was dose–effect relationships between biomarkers and mixtures levels of the various VOCs and CO	[110]
*In vivo*	Mice—Kunming	VOCs (formaldehyde, benzene, toluene, and xylene)	oxidative stress, GSH, MDA and T-AOC, total OS, and iNOS	There was a significant increase in ROS and MDA and TNOS and iNOS activities were also significantly higher in certain VOC mixtures	[93]
*In vivo*	Mice—Kunming	VOCs (formaldehyde, benzene, toluene, and xylene)	ROS and GSH	Intracellular ROS levels were increased along with increasing VOCs concentration	[94]
*In vivo*	Mice—C57B/L6	Air fresheners (VOCs)	SOD1, SOD2, and COX-2 expression	Induction of increased expression of SOD1, SOD2 and COX-1 in the mice fed the high fat diet, but not the normal diet once exposed to the air freshener	[114]
*In vivo*	Rat—Wistar	Formaldehyde	Nitrites in BAL cells	Significantly increased nitrites release in the formaldehyde group, which was increased after OVA exposure	[139]
*In vivo*	Rat—Wistar	Cooking oils	ROS, MDA, and GSH	ROS and MDA were significantly increased and GSH was decreased after the exposures (2 and 20 mg/kg)	[63]

CO, carbon monoxide; BaP-d12, benzo[a]pyrene-d12; BkF-d12, benzo[k]fluoranthene-d12; BghiP-d12, benzo[ghi]perylene-d12; DalP, dibenzo[a,l]pyrene; SB, strand breaks; 2,4,5-TCP, 2,4,5-trichlorophenol; PCP, pentachlorophenol; 4,6-DCG, 4,6-dichloroguaiacol; TeCG, tetrachloroguaiacol; 4,5-DCC, 4,5-dichlorocatechol; TeCC, tetrachlorocatechol; MN assay, micronucleus assay; VOCs, volatile organic compounds; A549s, human epithelial cell lines; THP-1, macrophage like cell lines; Ref, reference.

Lung cell types in the *in vitro* models have been seen to influence the concentration of ROS production after exposure to formaldehyde, with Calu-3 producing more ROS than 16HBE14o-. However, significant increases in ROS were identified after exposure to formaldehyde regardless of cell type (1400–7000 μM) [90] and other VOCs [91, 92] in *in vitro* models. This increase was also identified within rodent *in vivo* models when exposed to various mixtures of VOCs and different VOC compositions [93, 94].

DNA damage-inducible transcript 4 (DDIT4) is a gene that links the mitochondria and ROS production [95], and its loss is known to increase DNA damage related apoptosis [96]. Hexanal (a known indoor VOC) has been identified as causing this decrease in the expression of DDIT4 [97], and therefore to confirm potential DNA from the inhalation of this VOC there would need to be further observed damage assays completed. MDA (a biological marker for OS) was significantly increased after exposure to various VOCs mixtures in mice, with some VOCs increasing the concentration of MDA measured more than others [93]. Telomeres are susceptible to the effects of ROS and therefore base lesions induced by 8-oxoG, shortening the length of the telomeres [98]. In the 2012 study by Senthilkumar *et al.*, polychlorinated biphenyls (PBCs) had the capacity to significantly reduce the activity of the enzyme telomerase in HaCat cells over a 48-day period, there was a 20% reduction in the length of the telomere by day 18 and 30 which increased to 40% at days 42 and 48 [99]. In skin cells, indoor VOCs have been identified as reducing the telomere after a certain number of exposure days as well as different PCBs inducing changes in length. This does not just have implications for DNA damage and SBs but also premature aging of the cell and environmental stress exposure [100].

ROS has been identified to increase Cox-2 expression [101], which is responsible for the production of prostaglandin E_2_ and correlates with MDA concentrations [102]. MDA is mutagenic in multiple cell types and is known to react with DNA to induce the generation of adducts (M_1_G) [102]. Anything that has the potential to increase ROS production then has the potential to induce M1-dG. This increase in M1-dG has been identified in two separate occupational exposure groups exposed to formaldehyde [103, 104]. It has also been linked to increased ROS production *in vitro* [90, 105], suggesting the potential for this VOC to not only cause increased ROS production within the cells, but adducts within DNA.

Formaldehyde exposure was found to increase intracellular NO as well as stimulating inducible nitric oxide synthase (iNOS) expression [106]. Inducible NOS plays a key role in inflammation and immune cell activation as it produces NO from L-arginine [107], and both iNOS and NO are involved in cell signalling and DNA damage pathways [108]. After exposure to various mixtures of VOCs, iNOS was identified as being increased after specific mixtures [93].

Total antioxidant capacity (TAC) takes into consideration this potential balancing act between the production of ROS and the antioxidant capability of the system. It is the efficiency of various antioxidants to limit the oxidative degradation of different compounds; however, there is sometimes confusion within the literature as to TAC and total antioxidant activity [109]. There is some evidence in rodent models that there is an increase in GSH after exposure to various VOCs in both a dose and mixture dependant manner [110].

The superoxide dismutase family catalyses superoxide compounds to hydrogen peroxide and molecular oxygen [111] and are essential antioxidant enzymes [112]. Superoxide dismutase 1 (SOD1) is localised mostly within the cytoplasm of the cell [113]. Whereas Superoxide dismutase 2 (SOD2) is localised within the mitochondria and defends against mitochondrial superoxide [111]. After exposure to VOCs associated with air fresheners, there was increased expression of both SOD1 and SOD2 in a murine model fed a high fat diet, but not those mice on a normal diet [114], indicating that diet influenced this response. SOD levels are decreased after different VOC exposures in both rodent and human models. The comet assay has also identified the *in vitro* genotoxic potentials of different VOCs (compared to each other) as well as concentration differences [115] capable of inducing a response.

The chromosome aberration test is one of the Organisation for Economic Co-Operation and Development (OECD) genetic toxicology test battery approaches used to assess potential genotoxicity. However, this is considered an older approach and therefore other assays may be deemed more appropriate. Chromosomal aberrations (CA) are commonly due to errors in cell divisions, with different time points they occur influencing the outcome, from malformations to stillbirths and genetic syndromes [116]. Significant increases in CA were identified in workers on a pathology ward exposed to formaldehyde when compared to the office worker control. This suggests that exposure to formaldehyde [117] has the potential to cause significant DNA damage, with the further potential for generational damage [118].

### Metals

Metals within IAP tend to be associated with PM (and what process is happening within the local environment), with certain metals associated with human health impacts [119]. Heavy metals are found within the indoor environment [120]; however, there is very little work completed on them as a component of indoor air and the potential for genotoxicity. Aluminium is one of the most abundant metals humans are exposed to [121], with the manufacturing process containing many steps. Depending on the stage of the manufacturing process a person was involved in has been identified as influencing the SOD concentrations within the workers [122] and potentially the genotoxicity of the process (Table 1).

### Gases

There are various gases present within the IAP cocktail, however, there has been limited work completed on them and their genotoxic potential, with the focus on radon (Table 3). The micronucleus assay has been utilised to determine the potential genotoxic potential of radon exposures. No correlation was found between radon exposures and micronucleus frequency, but it did identify a gender difference in micronucleus frequency [123]. The micronucleus frequency is known to increase with age and is higher in females than males, and should always be considered when utilising this assay and comparing different population peripheral blood lymphocytes [124]. For exposures of radon, the DNA damage identified with the comet assay was FPG-related [123].

**Table 3:** Table summarising the genotoxicity of gases found within indoor air.

Model type		Component	Endpoint analysed	Outcome	Ref
*In vitro*	*A549*	NO	NO production	Significant increase in NO production after exposure to gaseous NO	[105]
Human study	Radon exposures	Radon	Comet assay	There was a significant increase in DNA damage associated with increased radon exposures	[123]
Human study	Biomass and LPG fuel use at home (Lucknow City, India)	gaseous components	MN assay	Elevated MN frequencies in cow dung, then cow dung/wood and then wood alone. Kerosene increased the frequency but not significantly	[79]
Human study	Radon exposures	Radon	MN assay	No significant correlation between MN frequency and radon concentration. There was a significant difference between males and females	[140]

NO, nitrogen oxide; LPG, liquid petroleum gas; MN assay, micronucleus assay; A549s, human epithelial cell lines; Ref, reference.

## What do we know?

To date, there has been limited investigation on the potential genotoxic capabilities of the various components of IAP, with PM, VOCs, and chemicals being the focus of most studies. It has been identified since as early as 2004 that mutagens are present within outdoor urban air [125] and 2006 that there is a link between outdoor air pollution and ^32^P-postlabelling analysis [126] for the detection of carcinogen–DNA adducts [127]. There is an established link between outdoor air pollution and genotoxicology using plants, *in vitro* models and human biomonitoring studies [128], including with children linking increased micronuclei frequency and high pollution concentrations [129]. As well as outdoor air (specifically PM_2.5_) being linked to driving mutations linked to lung cancer [130]. There are suggestions from the current review that these links and increases in genotoxic markers are also apparent from specifically indoor air pollutants.

What has been done highlights the potential for IAP components to cause induction of ROS and oxidative reactions as well as reacting directly on the DNA. With increasing time spent indoors and awareness of the toxicity of these components, it is important to identify the genotoxic potential. This will potentially allow for risk mitigation to be implemented within specific demographics of people (e.g. women cooking with biomass fuels). One area of air pollution genotoxicity that is currently completely missing from the literature is the exposure of various biologicals found indoors. Moulds, other fungi, and allergens are an emerging health risk and are of public interest. Little is known about the inhalation toxicology and mechanisms of action of these components, and this is becoming increasing important to understand due to public deaths linked to them. Most of the work focuses on biomass cooking and very specific demographic and economic populations. Further work is required to identify other components of indoor environments that may contribute towards the potential genotoxic risk. This includes the room types, the occupiers of the room, where the room is (outdoor environment), conditions of the room (humidity, ventilation, and temperature), and the use of the room (woodburning, candles, bathing, cleaning, and cooking (gas, electric, induction, and so on)—all having the potential to be influenced by the demographics of the users). This problem is as diverse as the people spending time in indoor environments.

Existing research on outdoor air pollution does not consider multi-pollutant exposures, assess exposures properly or consider potential differences in biological mechanisms [131], something that is also not considered in the indoor environment. Therefore, it is important to start addressing these gaps and further establishing the links between air pollution (both indoor and outdoor) and the potential for genotoxic and mutagenic effects from the air we breathe every day.

## Conclusion and outlook

We have begun to understand the genotoxic implications of the indoor environment with a heavy focus on chemicals on VOCs; however, there is still much that is unknown about the implications of breathing the air within certain environments. Limitations of the various studies that look at the indoor environment and the potential induction of genotoxic include only focusing on a single pollutant when that is not how humans are exposed to them. There are also not detailed measurements of actual concentrations within the air, and many studies use surrogate measurements to determine an outcome. Due to the complex nature of the indoor environment pollution mixture, there cannot be a certainty that what has specifically been measured within a study is the main influence of the biological outcomes. This is especially the case with the human *in vivo* studies, where elements (such as PM or VOCs individually) are measured, but not the complete mixture of pollutants. Individual component (potential) genotoxicity has been repeatedly overlooked, as well as mixtures and combinations in already at-risk individuals. This should be a focus for future research for the potential implications of breathing these pollutants to be assessed fully.

## Supplementary Material

Supplementary_Information_geag024

## Data Availability

The data underlying this article are available in the article and in its online supplementary material.

## References

[1] Kuehn BM . WHO: more than 7 million air pollution deaths each year. *JAMA* 2014;311:1486. 10.1001/jama.2014.4031 24737355

[2] Miller MR, Shaw CA, Langrish JP. From particles to patients: oxidative stress and the cardiovascular effects of air pollution. *Futur Cardiol* 2012;8:577–602. 10.2217/fca.12.43 22871197

[3] UK Health Security Agency . Statement on the differential toxicity of particulate matter according to source or constituents: 2022. In COMEAP. 2022. Date accessed 15 December 2025.

[4] Li T, Yu Y, Sun Z et al. A comprehensive understanding of ambient particulate matter and its components on the adverse health effects based from epidemiological and laboratory evidence. *Part Fibre Toxicol* 2022;19:67. 10.1186/s12989-022-00507-5 PMC9707232 36447278

[5] Lei R, Wei Z, Chen M et al. Aging effects on the toxicity alteration of different types of organic aerosols: a review. *Curr Pollut Rep* 2023;9:590–601.

[6] Offer S, Hartner E, Di Bucchianico S et al. Effect of atmospheric aging on soot particle toxicity in lung cell models at the air-liquid interface: differential toxicological impacts of biogenic and anthropogenic secondary organic aerosols (SOAs). *Environ Health Perspect* 2022;130:27003. 10.1289/EHP9413 PMC8812555 35112925

[7] Schmitz R . Modelling of air pollution dispersion in Santiago de Chile. *Atmos Environ* 2005;39:2035–47.

[8] Schaefer K, Emeis S, Hoffmann H et al. Influence of mixing layer height upon air pollution in urban and sub-urban areas. *Meteorol Z* 2006;15:647–58.

[9] Marcazzan GM, Vaccaro S, Valli G et al. Characterisation of PM10 and PM2.5 particulate matter in the ambient air of Milan (Italy). *Atmos Environ* 2001;35:4639–50.

[10] Hoek G, Brunekreef B, Goldbohm S et al. Association between mortality and indicators of traffic-related air pollution in the Netherlands: a cohort study. *Lancet* 2002;360:1203–9. 10.1016/S0140-6736(02)11280-3 12401246

[11] Chen F, Zhang W, Mfarrej MFB et al. Breathing in danger: understanding the multifaceted impact of air pollution on health impacts. *Ecotoxicol Environ Saf* 2024;280:116532. 10.1016/j.ecoenv.2024.116532 38850696

[12] Robertson S, Miller MR. Ambient air pollution and thrombosis. *Part Fibre Toxicol* 2018;15:1. 10.1186/s12989-017-0237-x PMC5753450 29298690

[13] Rider CF, Carlsten C. Air pollution and DNA methylation: effects of exposure in humans. *Clin Epigenetics* 2019;11:131. 10.1186/s13148-019-0713-2 PMC6724236 31481107

[14] Huang H-B, Lai C-H, Chen G-W et al. Traffic-related air pollution and DNA damage: a longitudinal study in Taiwanese traffic conductors. *PLoS One* 2012;7:e37412. 10.1371/journal.pone.0037412 PMC3357412 22629390

[15] Maciel-Ruiz JA, Reynoso-Noverón N, Rodríguez-Moreno DA et al. Geographical approach analysis of the impact of air pollution on newborn intrauterine growth and cord blood DNA damage in Mexico City. *J Expo Sci Environ Epidemiol* 2024;34:907–16. 10.1038/s41370-023-00618-x PMC11446826 38086972

[16] Klepeis NE, Nelson WC, Ott WR et al. The National Human Activity Pattern Survey (NHAPS): a resource for assessing exposure to environmental pollutants. *J Expo Anal Environ Epidemiol* 2001;11:231–52. 10.1038/sj.jea.7500165 11477521

[17] Abdalla TE, Peng C. Evaluation of housing stock indoor air quality models: a review of data requirements and model performance. *J Build Eng* 2021;43:102846.

[18] Tran VV, Park D, Lee YC. Indoor air pollution, related human diseases, and recent trends in the control and improvement of indoor air quality. *Int J Environ Res Public Health* 2020;17:2927. 10.3390/ijerph17082927 PMC7215772 32340311

[19] Rosário Filho NA, Urrutia-Pereira M, D'Amato G et al. Air pollution and indoor settings. *World Allergy Organ J* 2021;14:100499. 10.1016/j.waojou.2020.100499 PMC7806792 33510831

[20] Eriksson A, Abera A, Malmqvist E et al. Characterization of fine particulate matter from indoor cooking with solid biomass fuels. *Indoor Air* 2022;32:e13143. 10.1111/ina.13143 PMC9828024 36437670

[21] Dawson JN, DiMonte KE, Griffin MJ et al. Ultrafine particles emitted through routine operation of a hairdryer. *Environ Sci Technol* 2021;55:8554–60. 10.1021/acs.est.0c08564 34105951

[22] Chen EY, Lin KT, Jung CC et al. Characteristics and influencing factors of airborne microplastics in nail salons. *Sci Total Environ* 2022;806:151472. 10.1016/j.scitotenv.2021.151472 34742808

[23] Canepari S, Astolfi ML, Drago G et al. PM(2.5) elemental composition in indoor residential environments and co-exposure effects on respiratory health in an industrial area. *Environ Res* 2023;216:114630. 10.1016/j.envres.2022.114630 36279913

[24] Eftekhari A, Fortenberry CF, Williams BJ et al. Continuous measurement of reactive oxygen species inside and outside of a residential house during summer. *Indoor Air* 2021;31:1199–216. 10.1111/ina.12789 PMC8396106 33484190

[25] Maung TZ, Bishop JE, Holt E et al. Indoor air pollution and the health of vulnerable groups: a systematic review focused on particulate matter (PM), volatile organic compounds (VOCs) and their effects on children and people with pre-existing lung disease. *Int J Environ Res Public Health* 2022;19:8752. 10.3390/ijerph19148752 PMC9316830 35886604

[26] Noorian Najafabadi SA, Sugano S, Bluyssen PM. Impact of carpets on indoor air quality. *Appl Sci* 2022;12:12989.

[27] Omolaoye TS, Skosana BT, Ferguson LM et al. Implications of exposure to air pollution on male reproduction: the role of oxidative stress. *Antioxidants (Basel)* 2024;13:64. 10.3390/antiox13010064 PMC10812603 38247488

[28] Peng H, Wang M, Wang Y et al. The association between indoor air pollution from solid fuels and cognitive impairment: a systematic review and meta-analysis. *Rev Environ Health* 2024;85–96. 10.1515/reveh-2023-0158 38413202

[29] Hussain MS, Gupta G, Mishra R et al. Unlocking the secrets: volatile organic compounds (VOCs) and their devastating effects on lung cancer. *Pathol Res Pract* 2024;255:155157. 10.1016/j.prp.2024.155157 38320440

[30] Radbel J, Rebuli ME, Kipen H et al. Indoor air pollution and airway health. *J Allergy Clin Immunol* 2024;154:835–46. 10.1016/j.jaci.2024.08.013 PMC12337040 39182629

[31] Kazensky L, Matković K, Gerić M et al. Impact of indoor air pollution on DNA damage and chromosome stability: a systematic review. *Arch Toxicol* 2024;98:2817–41. 10.1007/s00204-024-03785-4 38805047

[32] Weschler CJ . Chemistry in indoor environments: 20 years of research. *Indoor Air* 2011;21:205–18. 10.1111/j.1600-0668.2011.00713.x 21281360

[33] Yang L, You B, Zhou W et al. Rapid evolution of indoor primary and secondary organic aerosols from human activities. *Build Environ* 2024;261:111736.

[34] UK Health Security Agency . Understanding and addressing the health risks of damp and mould in the home. In:*Ministry of Housing, Communities & Local Government*, 2024. Date accessed 12 March 2026.

[35] Hartiala M, Elenius V, Pesquera AA et al. Exposures in indoor air affecting health. *Allergy* 2026;81:700–19. 10.1111/all.70179 PMC12954573 41342236

[36] Won W-S, Noh J, Oh R et al. Enhancing the reliability of particulate matter sensing by multivariate Tobit model using weather and air quality data. *Sci Rep* 2023;13:13150. 10.1038/s41598-023-40468-z PMC10423292 37573439

[37] Park HJ, Lee HY, Suh CH et al. The effect of particulate matter reduction by indoor air filter use on respiratory symptoms and lung function: a systematic review and meta-analysis. *Allergy Asthma Immunol Res* 2021;13:719–32. 10.4168/aair.2021.13.5.719 PMC8419638 34486257

[38] Jones ER, Cedeño Laurent JG, Young AS et al. The effects of ventilation and filtration on indoor PM2.5 in office buildings in four countries. *Build Environ* 2021;200:107975. 10.1016/j.buildenv.2021.107975 PMC8336933 34366550

[39] Zani C, Donato F, Ceretti E et al. Genotoxic activity of particulate matter and In vivo tests in children exposed to air pollution. *Int J Environ Res Public Health* 2021;18:5345. 10.3390/ijerph18105345 PMC8156021 34067860

[40] Karlsson HL, Nygren J, Möller L. Genotoxicity of airborne particulate matter: the role of cell–particle interaction and of substances with adduct-forming and oxidizing capacity. *Mutat Res* 2004;565:1–10. 10.1016/j.mrgentox.2004.07.015 15576234

[41] Lee JW, Kim JS, Lee HJ et al. Age and gender effects on genotoxicity in diesel exhaust particles exposed C57BL/6 mice. *Biomolecules* 2021;11:374. 10.3390/biom11030374 PMC7998454 33801497

[42] Chatterjee N, Walker GC. Mechanisms of DNA damage, repair, and mutagenesis. *Environ Mol Mutagen* 2017;58:235–63. 10.1002/em.22087 PMC5474181 28485537

[43] Dilger M, Orasche J, Zimmermann R et al. Toxicity of wood smoke particles in human A549 lung epithelial cells: the role of PAHs, soot and zinc. *Arch Toxicol* 2016;90:3029–44. 10.1007/s00204-016-1659-1 26838041

[44] Ke S, Liu Q, Zhang X et al. Cytotoxicity analysis of biomass combustion particles in human pulmonary alveolar epithelial cells on an air-liquid interface/dynamic culture platform. *Part Fibre Toxicol* 2021;18:31. 10.1186/s12989-021-00426-x PMC8379799 34419099

[45] López M, López-Lilao A, Romero F et al. Size-resolved chemical composition and toxicity of particles released from refit operations in shipyards. *Sci Total Environ* 2023;880:163072. 10.1016/j.scitotenv.2023.163072 36990238

[46] Skovmand A, Damiao Gouveia AC, Koponen IK et al. Lung inflammation and genotoxicity in mice lungs after pulmonary exposure to candle light combustion particles. *Toxicol Lett* 2017;276:31–8. 10.1016/j.toxlet.2017.04.015 28465192

[47] Martikainen MV, Tossavainen T, Täubel M et al. Toxicological and microbiological characterization of cow stable dust. *Toxicol in Vitro* 2021;75:105202. 10.1016/j.tiv.2021.105202 34166725

[48] Jantzen K, Møller P, Karottki DG et al. Exposure to ultrafine particles, intracellular production of reactive oxygen species in leukocytes and altered levels of endothelial progenitor cells. *Toxicology* 2016;359-360:11–8. 10.1016/j.tox.2016.06.007 27311922

[49] Jeon MS, Kim JW, Han YB et al. Polystyrene microplastic particles induce autophagic cell death in BEAS-2B human bronchial epithelial cells. *Environ Toxicol* 2023;38:359–67. 10.1002/tox.23705 36485005

[50] Dutta A, Ray MR, Banerjee A. Systemic inflammatory changes and increased oxidative stress in rural Indian women cooking with biomass fuels. *Toxicol Appl Pharmacol* 2012;261:255–62. 10.1016/j.taap.2012.04.004 22521606

[51] Mukherjee B, Dutta A, Roychoudhury S et al. Chronic inhalation of biomass smoke is associated with DNA damage in airway cells: involvement of particulate pollutants and benzene. *J Appl Toxicol* 2013;33:281–9. 10.1002/jat.1748 22131134

[52] Mukherjee B, Bindhani B, Saha H et al. Increased oxidative DNA damage and decreased expression of base excision repair proteins in airway epithelial cells of women who cook with biomass fuels. *Environ Toxicol Pharmacol* 2014;38:341–52. 10.1016/j.etap.2014.06.010 25128766

[53] Chau L-Y . Heme oxygenase-1: emerging target of cancer therapy. *J Biomed Sci* 2015;22:22. 10.1186/s12929-015-0128-0 PMC4380252 25885228

[54] Otterbein LE, Hedblom A, Harris C et al. Heme oxygenase-1 and carbon monoxide modulate DNA repair through ataxia-telangiectasia mutated (ATM) protein. *Proc Natl Acad Sci* 2011;108:14491–6. 10.1073/pnas.1102295108 PMC3167547 21849621

[55] Ito T, Suzuki T, Tamura K et al. Examination of mRNA expression in rat hearts and lungs for analysis of effects of exposure to concentrated ambient particles on cardiovascular function. *Toxicology* 2008;243:271–83. 10.1016/j.tox.2007.10.013 18077076

[56] Khoubnasabjafari M, Ansarin K, Jouyban A. Reliability of malondialdehyde as a biomarker of oxidative stress in psychological disorders. *Bioimpacts* 2015;5:123–7. 10.15171/bi.2015.20 PMC4597159 26457249

[57] Ma B, Villalta PW, Balbo S et al. Analysis of a malondialdehyde–deoxyguanosine adduct in human leukocyte DNA by liquid chromatography nanoelectrospray–high-resolution tandem mass spectrometry. *Chem Res Toxicol* 2014;27:1829–36. 10.1021/tx5002699 PMC4203394 25181548

[58] Marnett LJ . Chemistry and biology of DNA damage by malondialdehyde. *IARC Sci Publ* 1999;150:17–27. 10626205

[59] Song P, Li Z, Li X et al. Transcriptome profiling of the lungs reveals molecular clock genes expression changes after chronic exposure to ambient air particles. *Int J Environ Res Public Health* 2017;14:90. 10.3390/ijerph14010090 PMC5295340 28106813

[60] Gauer B, Sauer E, Nascimento S et al. Cellular response to chemicals present in air pollution in occupationally exposed workers and its potential cancer susceptibility. *Chemosphere* 2021;263:127857. 10.1016/j.chemosphere.2020.127857 32854004

[61] Labarrere CA, Kassab GS. Glutathione: a Samsonian life-sustaining small molecule that protects against oxidative stress, ageing and damaging inflammation. *Front Nutr* 2022;9:1007816. 10.3389/fnut.2022.1007816 PMC9664149 36386929

[62] García-Giménez JL, Markovic J, Dasí F et al. Nuclear glutathione. *Biochim Biophys Acta* 2013;1830:3304–16. 10.1016/j.bbagen.2012.10.005 23069719

[63] Ma Y, Deng L, Ma P et al. In vivo respiratory toxicology of cooking oil fumes: evidence, mechanisms and prevention. *J Hazard Mater* 2021;402:123455. 10.1016/j.jhazmat.2020.123455 32683156

[64] De Bont R, van Larebeke N. Endogenous DNA damage in humans: a review of quantitative data. *Mutagenesis* 2004;19:169–85. 10.1093/mutage/geh025 15123782

[65] Burney S, Caulfield JL, Niles JC et al. The chemistry of DNA damage from nitric oxide and peroxynitrite. *Mutat Res* 1999;424:37–49. 10.1016/s0027-5107(99)00006-8 10064848

[66] Roy R, Jan R, Joshi U et al. Characterization, pro-inflammatory response and cytotoxic profile of bioaerosols from urban and rural residential settings in Pune, India. *Environ Pollut* 2020;264:114698. 10.1016/j.envpol.2020.114698 PMC7190302 32387676

[67] OECD . Test No. 489: In Vivo Mammalian Alkaline Comet Assay. OECD Guidelines for the Testing of Chemicals, Section 4. Paris: OECD Publishing, 2016. Date accessed 23 November 2020.

[68] Ladeira C, Møller P, Giovannelli L et al. The comet assay as a tool in human biomonitoring studies of environmental and occupational exposure to chemicals-a systematic scoping review. *Toxics* 2024;12:270. 10.3390/toxics12040270 PMC11054096 38668493

[69] Danielsen PH, Møller P, Jensen KA et al. Oxidative stress, DNA damage, and inflammation induced by ambient air and wood smoke particulate matter in human A549 and THP-1 cell lines. *Chem Res Toxicol* 2011;24:168–84. 10.1021/tx100407m 21235221

[70] Karlsson HL, Nilsson L, Möller L. Subway particles are more genotoxic than street particles and induce oxidative stress in cultured human lung cells. *Chem Res Toxicol* 2005;18:19–23. 10.1021/tx049723c 15651844

[71] Rodríguez-Carrillo A, Mustieles V, Salamanca-Fernández E et al. Implementation of effect biomarkers in human biomonitoring studies: a systematic approach synergizing toxicological and epidemiological knowledge. *Int J Hyg Environ Health* 2023;249:114140. 10.1016/j.ijheh.2023.114140 36841007

[72] Pandey AK, Bajpayee M, Parmar D et al. DNA damage in lymphocytes of rural Indian women exposed to biomass fuel smoke as assessed by the comet assay. *Environ Mol Mutagen* 2005;45:435–41. 10.1002/em.20106 15662701

[73] Jantzen K, Jensen A, Kermanizadeh A et al. Inhalation of house dust and ozone alters systemic levels of endothelial progenitor cells, oxidative stress, and inflammation in elderly subjects. *Toxicol Sci* 2018;163:353–63. 10.1093/toxsci/kfy027 29767793

[74] Laursen KR, Christensen NV, Mulder FA et al. Airway and systemic biomarkers of health effects after short-term exposure to indoor ultrafine particles from cooking and candles – a randomized controlled double-blind crossover study among mild asthmatic subjects. *Part Fibre Toxicol* 2023;20. 10.1186/s12989-023-00537-7 PMC10332087 37430267

[75] Sommer S, Buraczewska I, Kruszewski M. Micronucleus assay: the state of art, and future directions. *Int J Mol Sci* 2020;21:1534. 10.3390/ijms21041534 PMC7073234 32102335

[76] Fenech M . Cytokinesis-block micronucleus cytome assay. *Nat Protoc* 2007;2:1084–104. 10.1038/nprot.2007.77 17546000

[77] Fenech M . Cytokinesis-block micronucleus cytome assay evolution into a more comprehensive method to measure chromosomal instability. *Genes (Basel)* 2020;11:1203. 10.3390/genes11101203 PMC7602810 33076531

[78] Mondal NK, Mukherjee B, Das D et al. Micronucleus formation, DNA damage and repair in premenopausal women chronically exposed to high level of indoor air pollution from biomass fuel use in rural India. *Mutat Res* 2010;697:47–54. 10.1016/j.mrgentox.2010.02.006 20156583

[79] Musthapa MS, Lohani M, Tiwari S et al. Cytogenetic biomonitoring of Indian women cooking with biofuels: micronucleus and chromosomal aberration tests in peripheral blood lymphocytes. *Environ Mol Mutagen* 2004;43:243–9. 10.1002/em.20018 15141363

[80] Fatahi M, Reddig A, Vijayalaxmi Friebe B et al. DNA double-strand breaks and micronuclei in human blood lymphocytes after repeated whole body exposures to 7T magnetic resonance imaging. *Neuroimage* 2016;133:288–93. 10.1016/j.neuroimage.2016.03.023 26994830

[81] Mah LJ, El-Osta A, Karagiannis TC. γH2AX: a sensitive molecular marker of DNA damage and repair. *Leukemia* 2010;24:679–86. 10.1038/leu.2010.6 20130602

[82] Karagiannis TC, El-Osta A. Double-strand breaks: signaling pathways and repair mechanisms. *Cell Mol Life Sci* 2004;61:2137–47. 10.1007/s00018-004-4174-0 PMC11138614 15338043

[83] Xu J, Zhang Q, Su Z et al. Genetic damage and potential mechanism exploration under different air pollution patterns by multi-omics. *Environ Int* 2022;170:107636. 10.1016/j.envint.2022.107636 36423397

[84] Chirino YI, Sánchez-Pérez Y, Osornio-Vargas AR et al. PM(10) impairs the antioxidant defense system and exacerbates oxidative stress driven cell death. *Toxicol Lett* 2010;193:209–16. 10.1016/j.toxlet.2010.01.009 20096756

[85] Zhen AX, Piao MJ, Hyun YJ et al. Diphlorethohydroxycarmalol attenuates fine particulate matter-induced subcellular skin dysfunction. *Mar Drugs* 2019;17:95. 10.3390/md17020095 PMC6410332 30717280

[86] Zhen AX, Piao MJ, Kang KA et al. Niacinamide protects skin cells from oxidative stress induced by particulate matter. *Biomol Ther (Seoul)* 2019;27:562–9. 10.4062/biomolther.2019.061 PMC6824628 31272139

[87] Dergham M, Lepers C, Verdin A et al. Prooxidant and proinflammatory potency of air pollution particulate matter (PM_2·5−0·3_) produced in rural, urban, or industrial surroundings in human bronchial epithelial cells (BEAS-2B). *Chem Res Toxicol* 2012;25:904–19. 10.1021/tx200529v 22404339

[88] Niu BY, Li WK, Li JS et al. Effects of DNA damage and oxidative stress in human bronchial epithelial cells exposed to PM(2.5) from Beijing, China, in winter. *Int J Environ Res Public Health* 2020;17:4874. 10.3390/ijerph17134874 PMC7369897 32640694

[89] Europe WHO Regional Office for., and World Health Organization . WHO Guidelines for Indoor Air Quality: Selected Pollutants1st edn. Geneva: World Health Organization, 2010. 23741784

[90] Kastner PE, Casset A, Pons F. Formaldehyde interferes with airway epithelium integrity and functions in a dose- and time-dependent manner. *Toxicol Lett* 2011;200:109–16. 10.1016/j.toxlet.2010.11.003 21087659

[91] Kim JS, Klösener J, Flor S et al. Toxicity assessment of air-delivered particle-bound polybrominated diphenyl ethers. *Toxicology* 2014;317:31–9. 10.1016/j.tox.2014.01.005 PMC3975599 24451063

[92] Pěnčíková K, Svržková L, Strapáčová S et al. In vitro profiling of toxic effects of prominent environmental lower-chlorinated PCB congeners linked with endocrine disruption and tumor promotion. *Environ Pollut* 2018;237:473–86. 10.1016/j.envpol.2018.02.067 PMC5908724 29518658

[93] Wang F, Li C, Liu W et al. Effect of exposure to volatile organic compounds (VOCs) on airway inflammatory response in mice. *J Toxicol Sci* 2012;37:739–48. 10.2131/jts.37.739 22863854

[94] Wang F, Li C, Liu W et al. Effects of subchronic exposure to low-dose volatile organic compounds on lung inflammation in mice. *Environ Toxicol* 2014;29:1089–97. 10.1002/tox.21844 23418084

[95] Ding F, Gao F, Zhang S et al. A review of the mechanism of DDIT4 serve as a mitochondrial related protein in tumor regulation. *Sci Prog* 2021;104:36850421997273. 10.1177/0036850421997273 PMC10455034 33729069

[96] Vadysirisack DD, Baenke F, Ory B et al. Feedback control of p53 translation by REDD1 and mTORC1 limits the p53-dependent DNA damage response. *Mol Cell Biol* 2011;31:4356–65. 10.1128/MCB.05541-11 PMC3209328 21896779

[97] Cho Y, Lim JH, Song MK et al. Toxicogenomic analysis of the pulmonary toxic effects of hexanal in F344 rat. *Environ Toxicol* 2017;32:382–96. 10.1002/tox.22242 26880647

[98] De Rosa M, Johnson SA, Opresko PL. Roles for the 8-oxoguanine DNA repair system in protecting telomeres from oxidative stress. *Front Cell Dev Biol* 2021;9:758402. 10.3389/fcell.2021.758402 PMC8640134 34869348

[99] Senthilkumar PK, Klingelhutz AJ, Jacobus JA et al. Airborne polychlorinated biphenyls (PCBs) reduce telomerase activity and shorten telomere length in immortal human skin keratinocytes (HaCat). *Toxicol Lett* 2011;204:64–70. 10.1016/j.toxlet.2011.04.012 PMC3109099 21530622

[100] Lulkiewicz M, Bajsert J, Kopczynski P et al. Telomere length: how the length makes a difference. *Mol Biol Rep* 2020;47:7181–8. 10.1007/s11033-020-05551-y PMC7561533 32876842

[101] Onodera Y, Teramura T, Takehara T et al. Reactive oxygen species induce Cox-2 expression via TAK1 activation in synovial fibroblast cells. *FEBS Open Bio* 2015;5:492–501. 10.1016/j.fob.2015.06.001 PMC4476901 26110105

[102] Marnett LJ, Basu AK, O'Hara SM et al. Reaction of malondialdehyde with guanine nucleosides: formation of adducts containing oxadiazabicyclononene residues in the base-pairing region. *J Am Chem Soc* 1986;108:1348–50.

[103] Bono R, Romanazzi V, Munnia A et al. Malondialdehyde-deoxyguanosine adduct formation in workers of pathology wards: the role of air formaldehyde exposure. *Chem Res Toxicol* 2010;23:1342–8. 10.1021/tx100083x PMC2924748 20707408

[104] Bono R, Munnia A, Romanazzi V et al. Formaldehyde-induced toxicity in the nasal epithelia of workers of a plastic laminate plant. *Toxicol Res (Camb)* 2016;5:752–60. 10.1039/c5tx00478k PMC6062010 30090386

[105] Chen JH, Lin HH, Chiang TA et al. Gaseous nitrogen oxide promotes human lung cancer cell line A549 migration, invasion, and metastasis via iNOS-mediated MMP-2 production. *Toxicol Sci* 2008;106:364–75. 10.1093/toxsci/kfn195 18796497

[106] Ma H, Lin J, Li L et al. Formaldehyde reinforces pro-inflammatory responses of macrophages through induction of glycolysis. *Chemosphere* 2021;282:131149. 10.1016/j.chemosphere.2021.131149 34470174

[107] Cinelli MA, Do HT, Miley GP et al. Inducible nitric oxide synthase: regulation, structure, and inhibition. *Med Res Rev* 2020;40:158–89. 10.1002/med.21599 PMC6908786 31192483

[108] McAdam E, Haboubi HN, Forrester G et al. Inducible nitric oxide synthase (iNOS) and nitric oxide (NO) are important mediators of reflux-induced cell signalling in esophageal cells. *Carcinogenesis* 2012;33:2035–43. 10.1093/carcin/bgs241 22826608

[109] Flieger J, Flieger W, Baj J et al. Antioxidants: classification, natural sources, activity/capacity measurements, and usefulness for the synthesis of nanoparticles. *Materials (Basel)* 2021;14:4135. 10.3390/ma14154135 PMC8347950 34361329

[110] Wang F, Fangfang Z, Guo X et al. Effects of volatile organic compounds and carbon monoxide mixtures on learning and memory, oxidative stress, and monoamine neurotransmitters in the brains of mice. *Toxicol Ind Health* 2018;34:178–87. 10.1177/0748233717747504 29506457

[111] Flynn JM, Melov S. SOD2 in mitochondrial dysfunction and neurodegeneration. *Free Radic Biol Med* 2013;62:4–12. 10.1016/j.freeradbiomed.2013.05.027 PMC3811078 23727323

[112] Xu J, Su X, Burley SK et al. Nuclear SOD1 in growth control, oxidative stress response, amyotrophic lateral sclerosis, and cancer. *Antioxidants (Basel)* 2022;11:427. 10.3390/antiox11020427 PMC8869091 35204309

[113] Zelko IN, Mariani TJ, Folz RJ. Superoxide dismutase multigene family: a comparison of the CuZn-SOD (SOD1), Mn-SOD (SOD2), and EC-SOD (SOD3) gene structures, evolution, and expression. *Free Radic Biol Med* 2002;33:337–49. 10.1016/s0891-5849(02)00905-x 12126755

[114] Kim S, Lee AY, Cho MH. Inhaled exposure to air fresheners aggravated liver injury in a murine model of nonalcoholic fatty acid liver disease. *Heliyon* 2021;7:e06452. 10.1016/j.heliyon.2021.e06452 PMC8010405 33817364

[115] Gminski R, Tang T, Mersch-Sundermann V. Cytotoxicity and genotoxicity in human lung epithelial A549 cells caused by airborne volatile organic compounds emitted from pine wood and oriented strand boards. *Toxicol Lett* 2010;196:33–41. 10.1016/j.toxlet.2010.03.015 20362040

[116] Queremel Milani DA, Tadi P. Genetics, Chromosome Abnormalities. Treasure Island (FL): StatPearls, 2024.

[117] Santovito A, Schilirò T, Castellano S et al. Combined analysis of chromosomal aberrations and glutathione S-transferase M1 and T1 polymorphisms in pathologists occupationally exposed to formaldehyde. *Arch Toxicol* 2011;85:1295–302. 10.1007/s00204-011-0668-3 21360194

[118] Raudsepp T, Chowdhary BP. Chromosome aberrations and fertility disorders in domestic animals. *Annu Rev Anim Biosci* 2016;4:15–43. 10.1146/annurev-animal-021815-111239 26884101

[119] Chen L-C, Maciejczyk P, Thurston D et al. Chapter 6 - metals and air pollution. In: Nordberg GF, Costa M (eds.), *Handbook on the Toxicology of Metals (Fifth Edition)*, pp. 137–82. New York, USA: Academic Press, 2022.

[120] Chu H, Liu Y, Xu N et al. Concentration, sources, influencing factors and hazards of heavy metals in indoor and outdoor dust: a review. *Environ Chem Lett* 2023;21:1203–30.

[121] Exley C . The toxicity of aluminium in humans. *Morphologie* 2016;100:51–5. 10.1016/j.morpho.2015.12.003 26922890

[122] Hałatek T, Trzcinka-Ochocka M, Matczak W et al. Serum Clara cell protein as an indicator of pulmonary impairment in occupational exposure at aluminum foundry. *Int J Occup Med Environ Health* 2006;19:211–23. 10.2478/v10001-006-0033-6 17402216

[123] K W, J O, P P et al. Residential exposure to radon and levels of histone γH2AX and DNA damage in peripheral blood lymphocytes of residents of Kowary city regions (Poland). *Chemosphere* 2020;247:125748. 10.1016/j.chemosphere.2019.125748 31954338

[124] Fenech M, Bonassi S. The effect of age, gender, diet and lifestyle on DNA damage measured using micronucleus frequency in human peripheral blood lymphocytes. *Mutagenesis* 2011;26:43–9. 10.1093/mutage/geq050 21164181

[125] Claxton LD, Matthews PP, Warren SH. The genotoxicity of ambient outdoor air, a review: Salmonella mutagenicity. *Mutat Res* 2004;567:347–99. 10.1016/j.mrrev.2004.08.002 15572287

[126] Phillips DH, Arlt VM. The 32P-postlabeling assay for DNA adducts. *Nat Protoc* 2007;2:2772–81. 10.1038/nprot.2007.394 18007613

[127] DeMarini DM, Claxton LD. Outdoor air pollution and DNA damage. *Occup Environ Med* 2006;63:227–9. 10.1136/oem.2005.025502 PMC2078081 16556739

[128] Vargas VMF, da Silva Júnior FMR, Silva PT et al. A comprehensive overview of genotoxicity and mutagenicity associated with outdoor air pollution exposure in Brazil. *J Toxic Environ Health, Part B* 2023;26:172–99. 10.1080/10937404.2023.2175092 36775848

[129] Acito M, Fatigoni C, Villarini M et al. Cytogenetic effects in children exposed to air pollutants: a systematic review and meta-analysis. *Int J Environ Res Public Health* 2022;19:6736. 10.3390/ijerph19116736 PMC9180689 35682315

[130] Hill W, Lim EL, Weeden CE et al. Lung adenocarcinoma promotion by air pollutants. *Nature* 2023;616:159–67. 10.1038/s41586-023-05874-3 PMC7614604 37020004

[131] Gao P . The human airborne exposome. *Nature*. *Health* 2026;1:26–34.

[132] Champion WM, Warren SH, Kooter IM et al. Mutagenicity- and pollutant-emission factors of pellet-fueled gasifier cookstoves: comparison with other combustion sources. *Sci Total Environ* 2020;739:139488. 10.1016/j.scitotenv.2020.139488 PMC7676151 32526531

[133] Madu FU, Agoro ES, Madu MC. Exhaled breath condensate markers of oxidative stress in male storekeepers of chemical stores in the Ariaria international market Aba Abia state Nigeria. *Toxicol Ind Health* 2022;38:801–9. 10.1177/07482337221133885 36261326

[134] Mori Y, Tanaka-Kagawa T, Tahara M et al. Species differences in activation of TRPA1 by resin additive-related chemicals relevant to indoor air quality. *J Toxicol Sci* 2023;48:37–45. 10.2131/jts.48.37 36599426

[135] Michałowicz J, Majsterek I. Chlorophenols, chlorocatechols and chloroguaiacols induce DNA base oxidation in human lymphocytes (in vitro). *Toxicology* 2010;268:171–5. 10.1016/j.tox.2009.12.009 20025924

[136] Cho Y, Song MK, Kim TS et al. DNA methylome analysis of saturated aliphatic aldehydes in pulmonary toxicity. *Sci Rep* 2018;8:10497. 10.1038/s41598-018-28813-z PMC6043580 30002397

[137] Waidyanatha S, Black SR, Witt KL et al. The common indoor air pollutant α-pinene is metabolised to a genotoxic metabolite α-pinene oxide. *Xenobiotica* 2022;52:301–11. 10.1080/00498254.2022.2070047 PMC9298155 35473450

[138] Wong JYY, Vermeulen R, Dai Y et al. Elevated urinary mutagenicity among those exposed to bituminous coal combustion emissions or diesel engine exhaust. *Environ Mol Mutagen* 2021;62:458–70. 10.1002/em.22455 PMC8511344 34331495

[139] dos Santos L, Franco A, Domingos HV et al. Reduced allergic lung inflammation in rats following formaldehyde exposure: long-term effects on multiple effector systems. *Toxicology* 2009;256:157–63. 10.1016/j.tox.2008.11.011 19071189

[140] Walczak K, Olszewski J, Domeradzka-Gajda K et al. Micronuclei frequency in peripheral blood lymphocytes and levels of anti-p53 autoantibodies in serum of residents of Kowary city regions (Poland) with elevated indoor concentrations of radon. *Mutat Res Genet Toxicol Environ Mutagen* 2019;838:67–75. 10.1016/j.mrgentox.2018.12.007 30678830

