# Supplementary material for: How genotoxic is the indoor air environment?
Source: Mutagenesis. 2026 Jun 23;41(6):360–72. doi: 10.1093/mutage/geag024 (PMC13711078; doi:10.1093/mutage/geag024)
Supplement: Supplementary file 1 [file supplementary_information_geag024.docx]

**Supplementary Information:**

**Methods**

This review was completed following the Preferred Reporting Items for Systematic review and Meta-Analysis Protocol (PRISMA) [1,2].

*Search and Screening Strategy and Eligibility*

The following databases were searched: MEDLINE via PubMed, EMBASE.com and Scopus at Elsevier. Searches were limited to a specific date range (1^st^ January 2003 – 31^st^ December 2023), as well as publications in the English language, using the search string “air pollution OR air pollutant OR air quality AND indoor AND toxicology”. This was a deliberately wide search string to capture publications that do not mention genotoxicology specifically but do mention initiating events of interest.

Once the articles from the three databases had been identified, the screening process was initiated with abstract screening and then on to full text screening (Figure SI 1). Within the “identification” stage we removed any articles that were not with the correct date range, written in English and were duplicates from the various databases utilised. The “Abstract screening” excluded any publications that were not primary studies (including review publications) or peer-reviewed (such as conference abstracts); utilised estimated air pollution measurements and not directly measured samples; anything including tobacco or vapes, or did not consider the impact in an indoor environment; publications that characterised the indoor environment but did not consider the biological or toxicological impact or those that used modelling within their studies; any publications that included ecological systems, non-human *in vitro* assessment and *ex vivo*; public perception pieces; and epidemiology studies (Figure SI 2). At the next stage of screening “Eligibility” all full text articles were then obtained with any publications that were not accessible to Swansea University; contained self-reporting questionnaires; and did not contain genotoxic related endpoints further excluded. This left the remaining publications within the “Final Selection” and the data extraction element of the review.

Figure SI 1. The PRISMA approach applied to this literature review.

The initial search identified 1679 publications, 28 of which removed due to the language, 451 removed due to date constraints and 269 removed due to duplications. Abstract Screening removed 720 publications and Eligibility screening removed 161 publications leaving 50 records as eligible.

*Data Extraction*

Standardised data extraction tables were complied within Microsoft Excel as well as a Quality Criteria [3-5] applied to each publication within the Final Selection group. This aimed to cover *in vitro*, *in vivo*, and human quality criteria.

Figure SI 2. The inclusion and exclusion criteria applied when completing the PRISMA approach.

1. Liberati A, Altman DG, Tetzlaff J *et al.* The PRISMA statement for reporting systematic reviews and meta-analyses of studies that evaluate healthcare interventions: explanation and elaboration. *Bmj* 2009;**339**:b2700.
2. Page MJ, McKenzie JE, Bossuyt PM *et al.* The PRISMA 2020 statement: an updated guideline for reporting systematic reviews. *J Clin Epidemiol* 2021;**134**:178–89.
3. Klimisch HJ, Andreae M, Tillmann U. A systematic approach for evaluating the quality of experimental toxicological and ecotoxicological data. *Regul Toxicol Pharmacol* 1997;**25**:1–5.
4. Siivola KM, Burgum MJ, Suárez-Merino B et al. A systematic quality evaluation and review of nanomaterial genotoxicity studies: a regulatory perspective. Part Fibre Toxicol 2022;19:59.
5. Fernández-Cruz ML, Hernández-Moreno D, Catalán J et al. Quality evaluation of human and environmental toxicity studies performed with nanomaterials – the GUIDEnano approach. Environmental Science: Nano 2018;5:381–97.
